# Traffic congestion and economic context: changes of spatiotemporal patterns of traffic travel times during crisis and post-crisis periods

**DOI:** 10.1007/s11116-021-10170-y

**Published:** 2021-01-26

**Authors:** Amparo Moyano, Marcin Stępniak, Borja Moya-Gómez, Juan Carlos García-Palomares

**Affiliations:** 1 Department of Civil Engineering, Universidad de Castilla-La Mancha (UCLM), Avda, Camilo José Cela s/n, 13071 Ciudad Real, Spain; 2 Transport, Infrastructure and Territory Research Group (T-GIS), Department of Geography, Universidad Complutense de Madrid (UCM), C/ Profesor Aranguren s/n, 28040 Madrid, Spain; 3 Transport Research Centre -TRANSyT-UPM, Universidad Politécnica Madrid (UPM), C/ Profesor Aranguren, 3, 28040 Madrid, Spain

**Keywords:** Urban accessibility, Traffic congestion, Spatiotemporal pattern, Economic crisis, GNSS

## Abstract

This paper aims to evaluate the impacts of the economic context on traffic congestion and its consequential effects on private vehicle accessibility. We conduct a long-term analysis of spatiotemporal traffic congestion patterns in Madrid (Spain), comparing two urban realms: the 2008 economic crisis and the following post-crisis situation. We apply TomTom Speed Profiles data to assess daily variations in traffic congestion and their changes between both periods, and Twitter data to capture spatial patterns of the daily pulse of the city. Increased traffic, a by-product of economic recovery, resulted in higher congestion, particularly during peak hours. Nevertheless, these changes are spatially uneven. In the city core, an increase in congestion is relatively temporally homogeneous, while in the peripheral suburban zones, there has been only a marginal increase in travel times. On the other hand, in the urban outskirts, increased traffic congestion is more severe but visibly different between north and south. These differences have strong social connotations: over 40% of the population experienced a dramatic increase in travel times (more than 25%) during peak hours. Moreover, low-income groups are more likely to live in the more affected southern districts, suffering most the negative consequences of increased congestion.

## Introduction

Traffic congestion management has always been a key issue for transport planning, especially in urban and metropolitan scales. When congestion occurs, traffic moves at lower speeds (increasing travel times), reduces accessibility, and affects how one can interact with available opportunities (Moya-Gómez 2018). Therefore, congestion is a problem linked directly to both land-use and transport systems and presents negative externalities such as environmental or economic issues (Marshall and Dumbaugh 2020) or problems related to employment, public health, among others (Higgins et al. 2018). It is also a phenomenon linked to travel behaviour and car use patterns, as it occurs when demand reaches and/or exceeds the capacity limits of the road infrastructure.

The scientific literature on transport studies has confirmed that the economic situation directly affects mobility patterns. Sobrino and Monzón (2014) found a direct relationship between the use of cars and the economic context, and stated that during periods of economic crisis, urban mobility patterns change, and usually, the kilometres travelled by private vehicles decrease significantly. Different studies in the transport literature have focused on the analysis of changes in urban mobility patterns during periods of economic decline. They show changes in household strategies to reduce their costs, especially in transportation (Cascajo et al. 2018). During an economic crisis, families usually choose to reduce the use of their car and the frequency of certain types of travel (Ulfarsson et al. 2015), mainly optional displacements (Papagiannakis et al., 2018). The reduction in private vehicle travel generally translates into an increase in the use of public transport, even in cases where the cost of tickets has increased (Efthymiou and Antoniou 2017). All these studies detail the impact of the economic crisis on the mobility model of each family or individual and the modal choice and transport alternatives. However, the scientific literature has not focused so extensively on the influence of the economic situation on private vehicle accessibility, especially during the post-crisis period.

The main objective of this paper is to analyse and evaluate the impact of the 2008 economic crisis on levels of traffic congestion and, therefore, on changes in accessibility by private vehicle in the Madrid metropolitan area in Spain, one of the countries most affected by the crisis.^1^ The daily variations of traffic congestion are analysed for two periods: first, during the crisis, when the effects of the economic situation led to a significant decrease in the use of cars; and second, in the following post-crisis period, when the situation had already started to recover in terms of the use of cars. Based on this comparative analysis, a spatial typology of the different areas in Madrid is obtained using a clustering technique based on travel times and income levels to identify the relation between traffic and socio-economic contexts. In the methods, we apply a spatiotemporal approach to assess travel times at different times of the day using two new data sources. First, historical information about road links’ speed profiles provided by TomTom allows for a detailed analysis of changes in travel times between particular areas within the metropolitan area (Moya-Gómez and Geurs 2018). Second, Twitter data provide insights about fluctuations of the city’s activity areas (activity hotspots) (García-Palomares et al. 2018; Moya-Gómez et al. 2018).

The remainder of this paper is structured as follows. Section 2 summarises the existing literature on traffic evaluation and Big Data sources in private vehicle accessibility studies. Section 3 describes the data and methodology. Section 4 shows the results regarding the temporal and spatial analysis of travel times and their influence on the definition of the typology of the different parts of the metropolitan area of Madrid. Finally, Sect. 5 presents the main conclusions of the study.

## Literature review

### Accessibility, congestion and economic growth

Traffic behaviour, especially information about congestion levels, is considered a key issue in transport management. For many years the scientific literature has analysed congestion from different approaches and considered different indexes, such as level of service, lane occupancy ratesssssss, queues and capacity adequacy, among others (Boarnet et al. 1998; Rao and Rao 2012). Although many studies have addressed different congestion indexes and the desired factors or attributes that should be considered, one of the most recurrent factors is travel time (Levinson and Lomax 1996). Travel time influences users’ decisions relating to route selection, modal choice and practitioners' decisions as it allows for an understanding of road infrastructure improvements. Travel time is one of the main indicators of congestion levels, and therefore is a crucial factor in transport planning.

In simple terms, congestion is when the number of vehicles trying to use a specific road link reaches or exceeds the capacity for which the traffic network was designed, and this situation induces increases in travel time, which result in traffic delays. However, congestion is a very complex phenomenon that generates impacts at different scales: these include first-order or micro-scale impacts to individuals on the vehicles, such as traffic delays, and second-order or macro-scale impacts which affect activities and/or the regional economy (Sweet 2011). Precisely, this two-scale approach leads us to a continuing debate about mobility and accessibility approaches of congestion analysis. Mobility enters accessibility calculations significantly in congestion measures, although it is a ‘mean’ rather than an ‘end’ (Grengs et al. 2010). Congestion is not only a one-off issue associated exclusively with transport systems, but is also a consequence of the distribution and intensity of land use and different social interactions, among others. Therefore, it is necessary to incorporate these components to identify other causes and effects of congestion and determine possible side effects that may call into question some measures adopted to solve congestion problems (Levine and Garb 2002).

Recent studies have addressed the relationship between traffic congestion and the economy, and have produced differing evidence regarding the former's effects on regional economic growth (Marshall and Dumbaugh 2020). On the one hand, several studies have empirically demonstrated that severe traffic congestion decreases employment and income growth (Hymel 2009; Sweet 2014a). Moreover, some of these studies have shown that congestion is detrimental not only to firms, but also to household income (Jin and Rafferty 2017), and that reducing traffic congestion will provide economic benefits in terms of increasing employment and income growth.

On the other hand, some recent papers have stated that traffic congestion certainly has negative impacts, but is also a by-product of economic activity and social interaction (Sweet 2014b). Congestion must not be viewed only as a cost to society because agglomerations of activities frequently give rise to traffic congestion (Mondschein and Taylor 2017). Precisely, agglomeration trends and localisation economy theory are very relevant for understanding congestion and economic growth links. By focusing on firms’ location, certain studies have found that new firms prioritise being located near same-industry firms because the access advantages of these areas of agglomeration outweigh the impedances of traffic congestion (Osman et al. 2019). However, some differences are found depending on the scale of congestion and the sector: as mentioned before, while localised congestion may be a proxy for amenities valued by many firms, regional congestion may be detrimental (Sweet 2014b), especially to office-based firms (Hou 2016). Many of these studies are based on accessibility analyses highlighting the importance of land use and destination attractiveness in transportation planning. Access to employment, for instance, is highly conditioned by travel time delays but also by proximity-based issues (Thomas et al. 2018). Similarly, studies about polycentrism-based policies show that, in general, they may reduce congestion levels, although maybe not accessibility levels. This would depend on the number of new sub-centres, among many other variables (Li et al. 2019).

Besides the differing impacts of congestion on economic growth, most studies agree on the relevance of household income and employment as key variables in congestion analyses (Jin and Rafferty 2017; Mondschein and Taylor 2017; Osman et al. 2019; Thomas et al. 2018). However, to measure and evaluate individuals’ exposure to congestion properly, consideration must be given not only to commuting (employment/jobs access) but also to the whole activity-travel pattern of individuals and households (Kim and Kwan 2019).

Furthermore, it is important to understand the impacts of congestion on economic growth and vice versa. There is a bidirectional causation as economic success can lead to traffic congestion, but when traffic congestion is sufficiently impactful, it has the potential to affect economic activity (Marshall and Dumbaugh 2020). For instance, Jin and Rafferty (2017) analysed the interrelationship between income, employment and congestion. The first two of these variables are positively associated with the third. Similarly, Mondschein and Taylor (2017) stated that trip-making spatial patterns are generally associated with income levels, showing that low average trip-making rates are associated more with low-income households and vice versa. Moreover, it is essential to highlight that not only the direct impacts of these socioeconomic variables on congestion but also the different effects of the economic cycles—recessions and recoveries—depending on households’ profile, could help us better understand variations in congestion levels. Depending on the income level and the kind of employment sectors, individuals and households are not equally affected by economic crises: for instance, low-skilled workers with lower salaries and temporary contracts were precisely the profile that suffered the most among employment losses during the 2008 financial crisis (Lallement 2011). On the other hand, highly educated middle-aged individuals are more resilient to economic cycles (Doran and Fingleton 2016).

Apart from these economic-related variables, congestion levels are both temporally and spatially influenced. Many studies on traffic congestion have focused on peak-hour periods during working days as the most relevant scenarios to be analysed. However, long-term spatial and temporal analysis is needed to fully understand congestion patterns for both commuting and non-commuting trips (Zhao and Hu 2019). As Weber and Kwan (2002, p 226) stated: ‘*the temporal dimension is very important to accurately assessing individual accessibility*’. Precisely, the next section addresses how new data sources are offering wider opportunities for travel time and congestion analysis from the spatio-temporal perspective.

### New data sources for measuring traffic travel times and congestion

Nowadays, the extensive use of different devices and the resulting data revolution have led to a new generation of interdisciplinary accessibility models. Measuring travel time and congestion now benefits from advances in geospatial technology and the availability of massive geo-located data, which are characterised by their high temporal and spatial resolution. As Geurs and Osth (2016, p 295) stated: ‘*It seems with advances in geospatial technology, internet technology, and growing abundance of detailed spatial data and real-time transport datasets, the field of accessibility modelling is thriving.*’ All this information offers many possibilities, especially for continuous accessibility analyses, which are based on examining temporal variations in accessibility (Chang and Cheon 2019; Zhao and Hu 2019), and are using real-time driving information, open-source mapping, and public transit supply data (Geurs and Östh 2016; Järv et al. 2018).

Concerning traffic, new applications using Floating Car Data (FCD), such as Google Maps Traffic Overlay, AutoNavi, Waze, or TomTom Live Traffic, show real-time traffic information for users and allow for the collection of information such as traffic volume, average traffic speed and actual journey times (Bartosiewicz and Wiśniewski 2015). Google Maps is the most extended application that can calculate optimal routes for different transport modes. Private vehicle travel times are based on tracks’ historical data combined with real-time traffic patterns from mobile phone records. Using Google API services, researchers can apply this data, computing OD travel time matrices for different times and days of the week (Dumbliauskas et al. 2017), which allows us to analyse the impacts of congestion in different temporal scenarios (García-Albertos et al. 2019). Also, thanks to GTFS files, API services allow interested parties to analyse the level of coverage of public transport networks, average speeds, and line overlaps (Hadas 2013), as well as to compute travel time matrices according to time slots, which can be used in dynamic accessibility studies (Boisjoly and El-Geneidy 2016; Fransen et al. 2015; Pritchard et al. 2019; Stępniak et al. 2019). AutoNavi is the largest Chinese web mapping, navigation, and location-based services provider that offers digital maps and real-time traffic information. Based on accumulated massive traffic and travel data of millions of AutoNavi map users, certain studies have identified it as a useful data source for measuring traffic congestion (Li et al. 2019). On the other hand, Waze is a mobility-oriented social network that allows users to obtain real-time traffic information, such as optimal routes, traffic speed, travel times, low-speed points, among others. As a contribution to this social network, users can also provide certain information related to traffic jams, car accidents, and road works, for example. All this information can be downloaded through Waze’s API and applied to specific urban studies from crowdsourced data related to traffic accidents (Angeles Perez et al. 2018; Santos et al. 2017).

TomTom provides detailed information about road networks and traffic and offers different products. One of them is the TomTom Multinet product, which is implemented in some studies both at the European(Ibáñez and Rotoli 2017), national (Moya-Gómez and Geurs 2018) or city levels (Schio et al. 2019). This database provides a homogenised network base for accessibility analyses. Then there is the useful TomTom Speed Profiles product, a digital network for private transport, which includes the average speeds of vehicles for each road link every five minutes. This historical data, obtained by different devices, including browsers and mobile phone GPS, enable dynamic accessibility analysis that considers the effects of congestion. In the literature, certain studies are now considering TomTom’s historical information (links’ speed profiles) in their quest to measure travel times at different times of the day or even to analyse the effects of link failure in transportation networks (Cui and Levinson 2017). Condeço-Melhorado et al. (2016) use the TomTom database to calculate internal travel distances for European NUTS-3 regions. Other studies are more oriented toward analysing daily variations in speed profiles for automobiles, which allows for an assessment of congestion impacts on accessibility (Moya-Gómez and García-Palomares 2015, 2017; Moyano et al. 2018) or analysing changes in spatial–temporal job accessibility during different periods (Moya-Gómez and Geurs 2018; Pritchard et al. 2019). Also, TomTom is now offering a new web service for developers, Traffic Stats,^2^ which collects FCD from TomTom navigation devices (in-dash systems and apps). These devices send anonymised data to TomTom servers in real-time and allow subscribers to derive historical traffic information.

In addition to network performance, the analysis of daily accessibility should also incorporate the effects of city dynamics (García-Palomares et al. 2018), a reason for congestion. Social networks such as Twitter or Flickr provide massive geo-located information about their users at different times of the day. The literature has used this information to understand and map specific mobility patterns (Luo et al. 2016; Salas-Olmedo and Rojas Quezada 2017). Moreover, this information reflects the temporal location of the main activity areas, in which there is a concentration of workers, tourists and/or residents, which can serve as a dynamic measure for a city’s activity hotspots. Accordingly, such dynamic data sources combined with other transport-oriented spatiotemporal information have become invaluable for their contribution to accessibility studies (Moya-Gómez et al. 2018; Moyano et al. 2018). However, the use of this spatial data in accessibility analysis is still in its early stages (Condeço-Melhorado et al. 2018), although its potential for transportation studies is considerable.

## Data and methods

Methodologically, for the Madrid case study we analyse traffic congestion variations and their effects on accessibility, using new data sources such as TomTom’s Historic Speed Profiles and Twitter data. In our study, we first propose the calculation of travel times throughout the day for comparisons between the two periods mentioned and, second, to develop a spatial typology of different areas of Madrid with similar profiles.

### Case study

This paper focuses on the Madrid metropolitan area, whose definition varies depending on the sources. In this paper, the delimitation used is based on a population density analysis. The metropolitan area is defined as the area composed of all the municipalities with more than 50% of their territory within a density isoline of 500 inhabitants/km2 from the central city (Moya-Gómez and García-Palomares 2015). The metropolitan area of Madrid consisted of 5.44 million inhabitants in 2008. In the years that followed, the population remained almost stable (5.57 million inhabitants in 2012). However, the subsequent economic recovery resulted in a more considerable population increase to 5.96 million inhabitants in 2017.

**Fig. 1:**
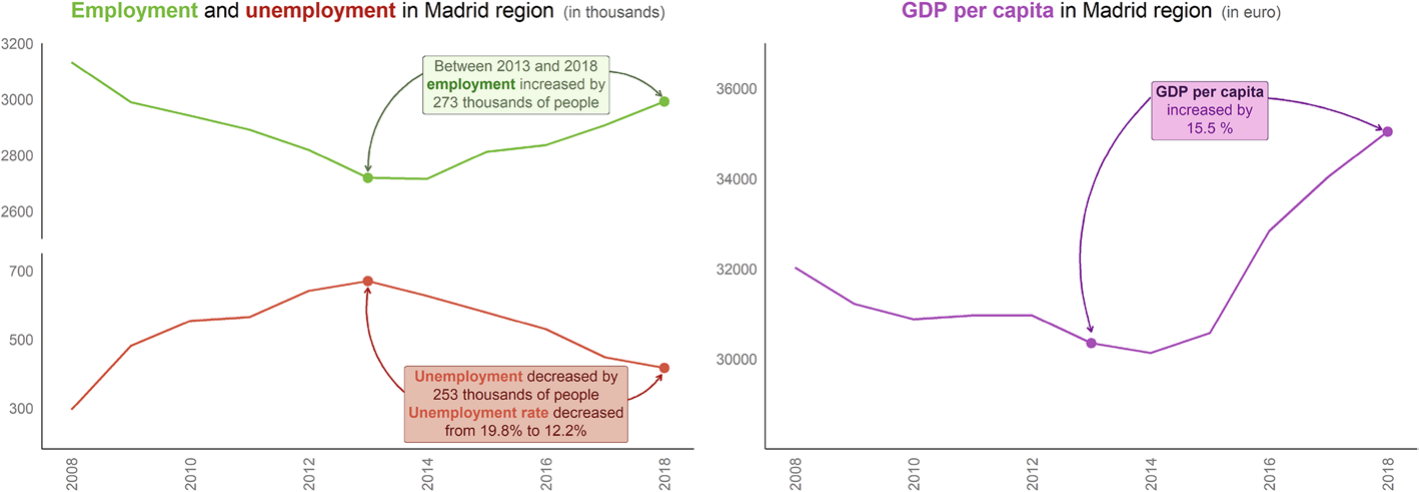
Main socio-economic indicators between 2008 and 2018 in the Madrid region

The effects of the 2008 economic crisis were significant in Spain and reached their nadir in 2012–2013. Madrid was strongly affected by the crisis: the GDP per capita decreased from €32,025 per inhabitant in 2008 to €30,349 in 2013, and then reached €35,041 per inhabitant in 2018. Also, the crisis in Madrid had a highly negative impact on employment rates: activity fell very sharply in the toughest years of the crisis, with unemployment affecting almost 20% of the working population between 2012 and 2013. However, by the end of 2017, this rate had already decreased to values similar to those before 2008 (Fig. 1). The generalised decrease in wealth and economic activity directly impacted mobility, with reduced congestion levels, especially at peak times because of a significant reduction of trips to work and the increase in the use of public transport (cheaper).

## Research design: travel times data and socio-economic variables

In this paper, we analyse the impact of the 2008 economic crisis in territorial accessibility by private vehicle because of the decrease in congestion. Accessibility is a dynamic attribute of locations that varies over time due to changes in the transport network and in the attractiveness of destinations for certain activities. To introduce this dynamic approach, we use a traditional, easy-to-understand accessibility indicator (weighted average travel times), which considers both the role of travel times and the relevance of the destinations to reach.

Travel time data were obtained from TomTom data, integrated into the ‘Network Analysis’^3^ toolbox in ArcGIS 10.3. This study used TomTom Historic Speed Profiles information for the period of economic crisis and the era of recuperation (2013 and 2018 versions of TomTom® data, respectively^4^). During these two periods, the structure of the data from TomTom changed slightly: there are some variations in the classification of the links composing the road network in Madrid. In this study, the links used are defined by TomTom as ranging from 0 to 6 in the Functional Road Classification (FRC), which is the attribute that indicates the type of road, based mainly on the characteristics of the roadway.^5^ The links defined as FRC 0 to 5 have historic speed profiles associated with more than 90% of the cases, both in 2013 and 2018. The FRC 6 group increased the percentage of links with a historical speed profile from 72% in 2013 to more than 90% in 2018. Although the 2018 TomTom data is more detailed, it does not have a crucial impact on accessibility analyses because the main improvements affect only local streets.

**Fig. 2:**
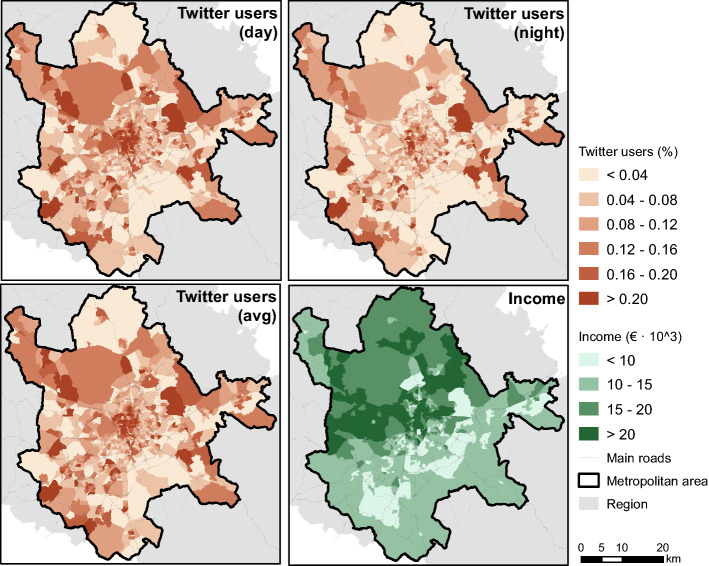
Twitter users and income level in the Madrid metropolitan area

Twitter data were used as the weight factor to introduce the importance/attractiveness of the destinations in the accessibility analysis. This data include all the free downloaded geo-located tweets registered in the Madrid study area from 2012 and 2013. Several reasons justify the selection of this variable and its reliability. Firstly, Twitter provides useful information about the whole activity-travel patterns of individuals throughout the city(Osorio-Arjona and García-Palomares 2019), which is a more accurate evaluation of individuals’ exposure to congestion than considering only commuting or labour trips (Kim and Kwan 2019). The work of Osorio-Arjona and García Palomares (2019) demonstrated that the level of precision offered by Twitter was adequate and efficient, permitting the analysis of flows between different zones of a specific city or study area. Second, Twitter data serve as a proxy of activity and provide sufficient disaggregation for application to different geographical areas, in our case, to Transport Zones.^6^ In addition, compared to other, more common weighted-factor variables such as population distribution, employment or income (Geurs and van Wee 2004), Twitter allows us to examine the daily variations in the attractiveness of different urban destinations. Twitter users can be considered a proxy for population/activity distribution in each Transport Zone (García-Palomares et al. 2018); the differences in the spatial distribution during the day and night times show cities’ hotspots at different times of the day. As Moya-Gómez et al. (2018) suggested, these daily variations are of great importance when analysing the impacts of congestion on urban accessibility. In Fig. 2, the spatial distribution of Twitter users registered during the period mentioned above is represented during the day (8:00–20:00 h) and night (22:00–6:00 h), and also the average value throughout the whole day in the study area. During the day, the northern and especially the central areas can be identified as activity centres, while dispersed zones both in the southeast and southwest maintain their activity levels during the night and can be identified mainly as residential areas.

In addition, the spatial distribution of the average annual income of the inhabitants in each Transport Zone^7^ is shown (Fig. 2). We can find remarkable differences between the north and south areas in the Madrid metropolitan area: in the north, the average income is higher than €15,000, and the west and northeast corridors are the wealthiest areas.

In this paper, we use the average income data to characterise the different parts of the metropolitan area. Income is a socio-economic variable commonly used in congestion analyses as it is representative in the bidirectional causation between congestion and economic growth widely mentioned in the literature (Jin and Rafferty 2017; Marshall and Dumbaugh 2020; Mondschein and Taylor 2017). More specifically, the crisis did not affect all social groups equally, nor consequently, the different urban areas. The economic recovery did not evolve at the same rate in different metropolitan areas and for different social groups. For these reasons, we consider average income as a variable to analyse how changes affect different spaces in the city.

### Methods

The proposed methodology is based on a computation of spatiotemporal measures of travel time every 15 min between Transport Zones during the day. The mass centres (considering population distribution) of all the 1171 Transport Zones included in the Madrid region are defined as origins/destinations for calculating travel times, although the specific results are obtained only for the metropolitan area. Then, a weighted average measure is calculated (${WTT}_{i}$) for each Transport Zone (1):

1 $${{WTT}_{i}}^{t}= \left(\sum_{j}{{t}_{ij}}^{t}\cdot {Tw}_{j} \right)/\sum_{j}{Tw}_{j}$$

where

- ${{t}_{ij}}^{t}$ is the travel time from origin Transport Zone *i* to destination *j*, departing at time *t*.
- ${Tw}_{j}$ is the mass factor represented by the average value of Twitter users during the whole day in the Transport Zone of destination *j* (see Fig. 2).

Once the computation parameters and variables are decided, the temporal variability of travel times is analysed in two different periods—crisis and post-crisis—and at four different times of the day: (1) Free Flow Speed (FFS) at 00:00 h, (2) morning peak hour at 8:00 h, 3) morning on-peak hour at 12:00 h, and 4) afternoon peak hour at 17:30. The complete analysis of travel times at different periods and times of the day allows us to perceive the differences in congestion impacts between the economic crisis and post-crisis periods.

Then, a spatiotemporal analysis is carried out, considering variations of traffic congestion and the share of the affected population. We take advantage of Twitter data and apply it as a proxy of the population’s spatial distribution at different times of the day. The character of Twitter data makes it a valuable data source regarding population distribution both in spatial and temporal dimensions.

Finally, considering the travel times analysis results, a typology of the different Transport Zones was carried out. In this analysis, a k-means cluster has been used to analyse the 1007 Transport Zones existing in Madrid metropolitan area. The k-means method is a non-hierarchical clustering technique that uses the Euclidean distance between items to establish groupings. Its objective is to minimise the distance among items in the same group and maximise distances among groups (Peña 2002). In the cluster analysis, we considered the following variables for each Transport Zone:

- *Free flow WTT*: is the value of weighted travel times at Free-Flow Speed (FFS) during the crisis period.
- *Relative change: 08:00*: is the ratio between WTTpost-crisis and WTTcrisis during the morning peak hour (8:00 h).
- *Relative change: 12:00*: is the ratio between WTTpost-crisis and WTTcrisis during the morning nonpeak hour (12:00 h).
- *Relative change: 17:30*: is the ratio between WTTpost-crisis and WTTcrisis during the afternoon peak hour (17:30 h).
- *Income level*: is the average value of annual income of the population in each Transport Zone (see Fig. 2).

The k-means cluster's computation was carried out using the ‘Grouping’ tool^8^ of the Spatial Statistical Package in ArcGIS 10.3. To understand the role or impact of each variable in the grouping process, R^2^ is computed as (TSS—ESS) / TSS; where TSS is the total sum of squares, and ESS is the explained sum of squares. TSS is calculated by squaring and then summing deviations from the global mean value for a variable. ESS is calculated the same way, except deviations are group by group: every value is subtracted from the mean value for the group it belongs to, then squared and summed. The R^2^ value reflects how much of the variation in the original variables was retained after the grouping process, so the larger the R^2^ value is for a particular variable, the better that variable is at discriminating among your features.

## Results

### Temporal variations of travel times

**Fig. 3:**
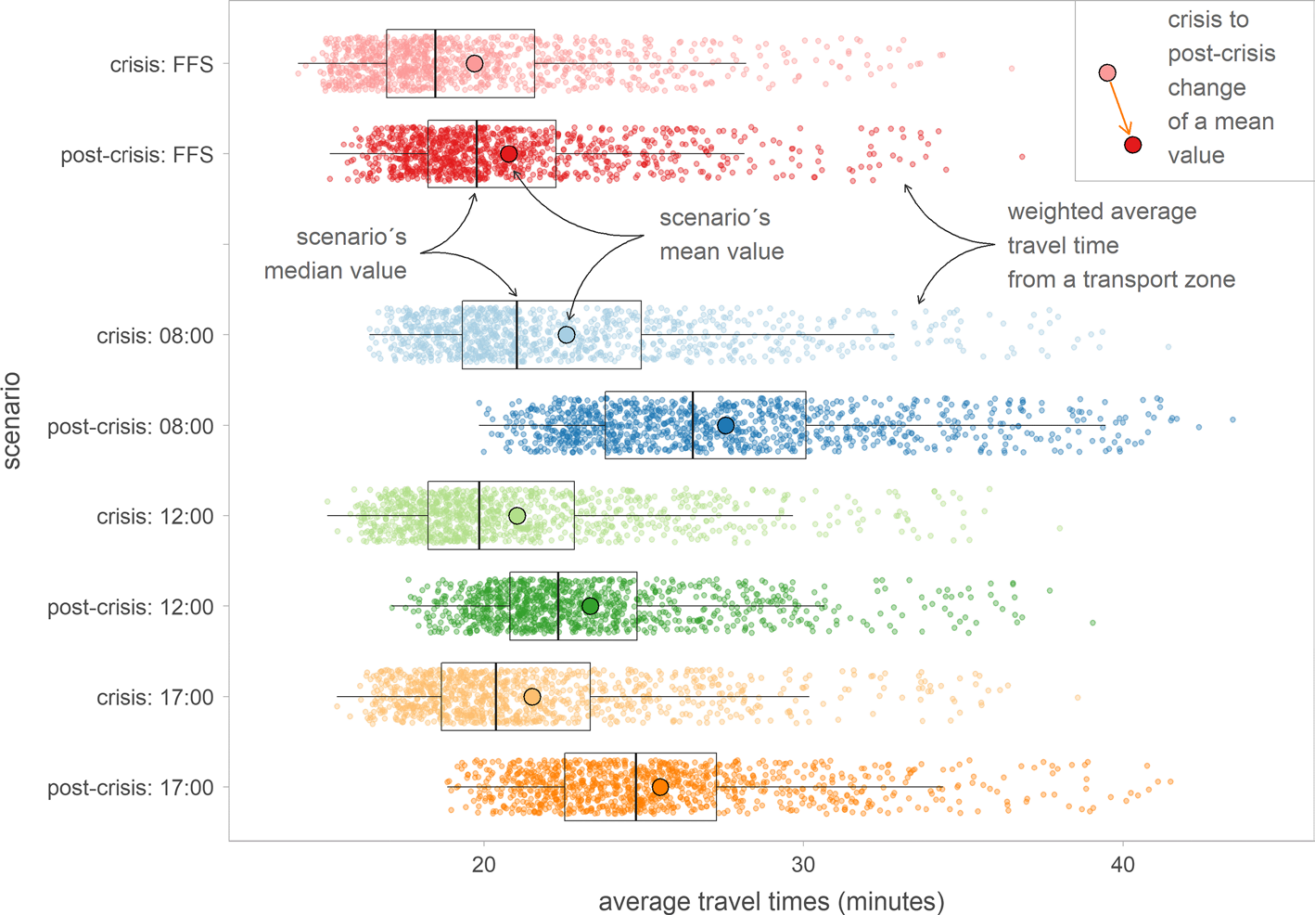
Boxplot analysis of travel times in the Madrid metropolitan area

In general, as considered in the previous hypothesis, the post-crisis situation presents longer travel times compared to the crisis period: in all the temporal scenarios considered, travel time mean values are higher in the post-crisis, especially during the morning and afternoon peak hours. As an average for the whole Madrilenian metropolitan area, travel times reach 27.9 min during the morning peak hour, 23.7 min during the morning off-peak hour and 25.9 min during the afternoon peak hour. This translates into increases of 21.6%, 10.7% and 18.3%, respectively, in comparison with the crisis period. In addition, and especially remarkable, is the increase in travel times in Free Flow Speed (FFS)—an average increase of 5.3% (Table 1 and Fig. 3). A priori, during the FFS scenario, travel times were expected to remain equal or even decrease due to the development of new road infrastructures. However, this increase was located mainly in the city’s central areas, where some streets were pedestrianised, forcing private vehicles to take longer detours (see in Fig. 3 and Table 1 that the minimum FFS difference is higher than the maximum one).

**Table 1:** General statistics of travel times in the crisis and post-crisis periods

	Crisis period				Post-crisis period				Difference post-crisis—crisis			
	FFS	8:00	12:00	17:30	FFS	8:00	12:00	17:30	FFS	8:00	12:00	17:30
Mean	20.1	22.9	21.4	21.9	21.2	27.9	23.7	25.9	1.1 (5.3%)	5 (21.6%)	2.3 (10.7%)	4.0 (18.3%)
Max	36.5	41.4	38.0	38.6	36.8	43.4	39.1	41.5	0.3 (0.9%)	2.0 (4.9%)	1.0 (2.7%)	2.9 (7.5%)
Min	14.2	16.4	15.1	15.4	15.2	19.9	17.1	18.9	1.0 (7.1%)	3.5 (21.0%)	2.0 (13.5%)	3.5 (22.5%)
SD	3.9	4.6	4.0	4.1	3.6	4.8	3.7	4.2	− 0.3	0.2	− 0.3	0.1
CV (%)	19.8	20.4	19.0	18.9	17.4	17.5	16.0	16.4	− 2.3	− 2.9	− 3.0	− 2.5

**Fig. 4:**
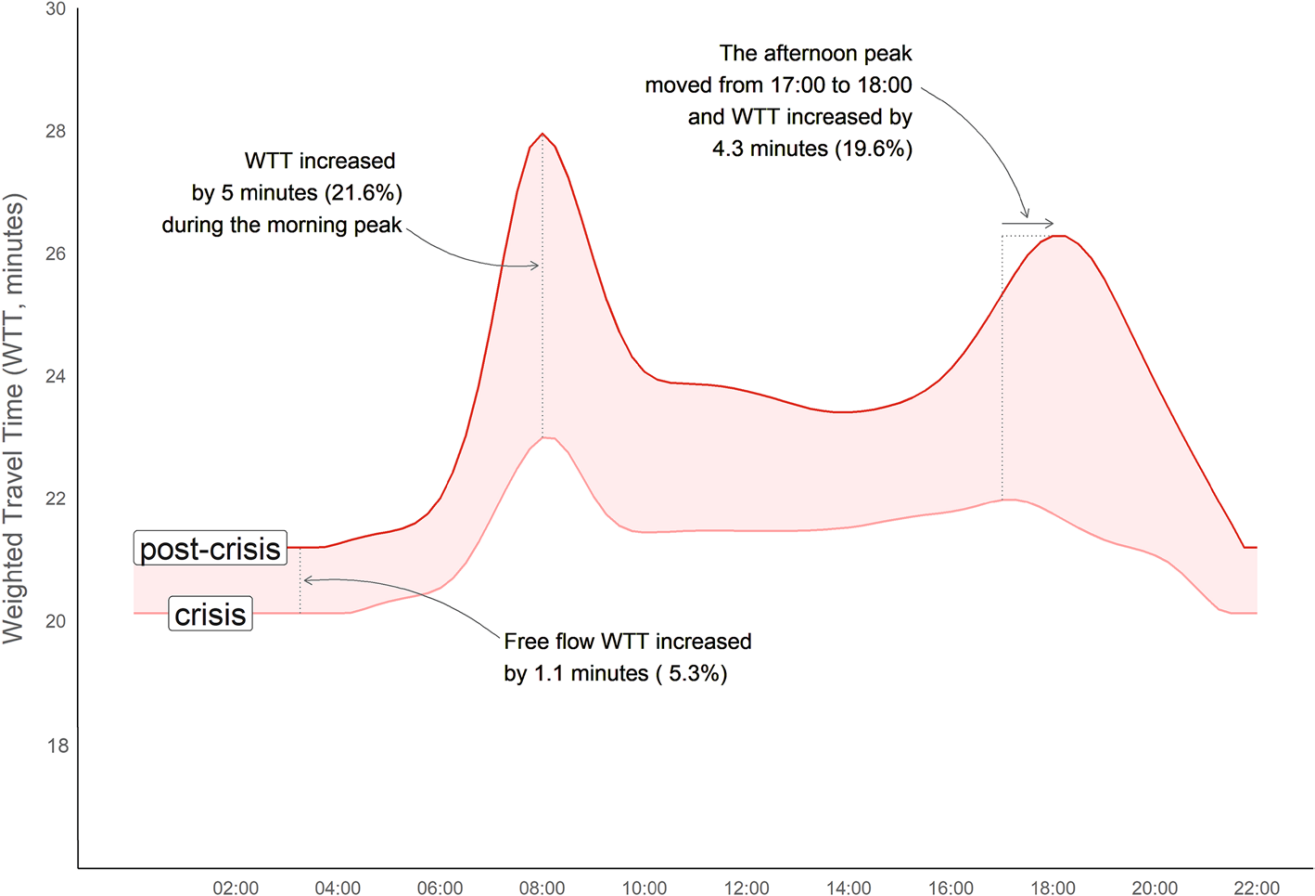
Weighted average travel times (WTT) in the crisis and post-crisis periods

In Fig. 4, the global average of these values in the Madrid metropolitan area is compared for each period. A more significant increase in congestion levels during rush hours in the post-crisis period can be observed than during the crisis, as mentioned above. However, this comparison allows us to detect not only a global travel time increase but also changes in the general trends of the graphs: the morning peak hour is clearly defined in the two periods, while the off-peak window and afternoon peak hour present variations between crisis and post-crisis situations. In the crisis, the minimum travel time during the day is experienced at 10:00 h and then increases continuously and slightly until the afternoon peak hour at 17:30, when it starts to decrease; however, during the post-crisis, a small increase in travel times is seen around 11:30 h, and then they decrease until the minimum daily value at 14:00 h. Also, the afternoon peak hour is later, at approximately 18:00 h, and a little longer than in the crisis scenario.

### Spatiotemporal analysis of congestion variations between crisis and post-crisis periods

**Fig. 5:**
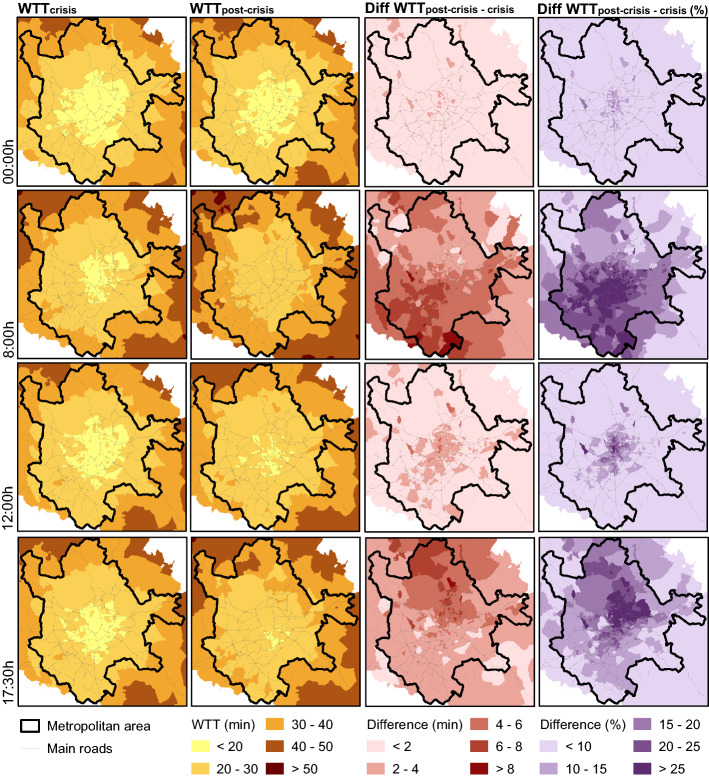
Weighted average travel times and differences between them in Madrid

In analysing these differences both temporally and spatially (Fig. 5), we find that travel times (WTT) in the crisis period are less than 20 min for the four times of the day in almost all Transport Zones in the city centre, while in post-crisis the central area increased its WTTs to between 20 and 30 min, except at 00:00 h in FFS. In addition, in the crisis period, the whole metropolitan area presents WTTs lower than 40 min, while in post-crisis, some parts in the north and north-west present higher travel time values in peak-hour scenarios.

Focusing on the differences shown in Fig. 5, this spatial analysis allows us to identify that higher increases in congestion are found in the south and south-west areas of Madrid during the morning peak hour (between 6 and 8 min increase) and in the north-east areas during the afternoon peak hour, reaching increases of more than 25% in both cases. Also, the central areas are the most affected during the off-peak scenarios, confirming the explanations made in Sect. 4.1 about the travel time increase in the FFS scenario.

**Fig. 6:**
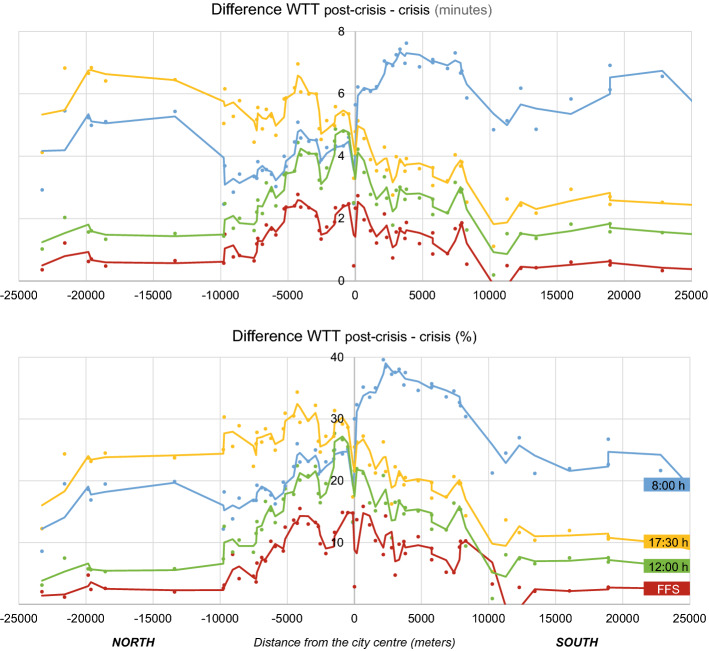
Absolute and relative differences of weighted travel times in the north–south cross section (measuring the Euclidean distance from the city centre)

Figure 6 shows cross-sections of congestion increase according to their Euclidean distance from the city centre at different times of the day, underlying the city's south-north division. During the morning peak hours, the most significant increase in travel time occurs in the southern part of the city, particularly between 2.5 and 7.5 km away from the city centre. On the contrary, an increase in afternoon congestion affects mostly the northern outskirts of the city, mainly around 5 km from the centre. Moreover, an increase in morning congestion is more severe than afternoon congestion (up to 40% vs less than a 30% increase, respectively). The areas most affected by an increase in congestion in the 12:00 h scenario are mainly downtown (cf. Figure 5), particularly within 2.5 km of the centre. In this first distance, we can observe the main differences between the off-peak and FFS scenarios, with an increase of more than two minutes of weighted travel time, while they differ by only one minute in the rest of the cross-section areas.

**Fig. 7:**
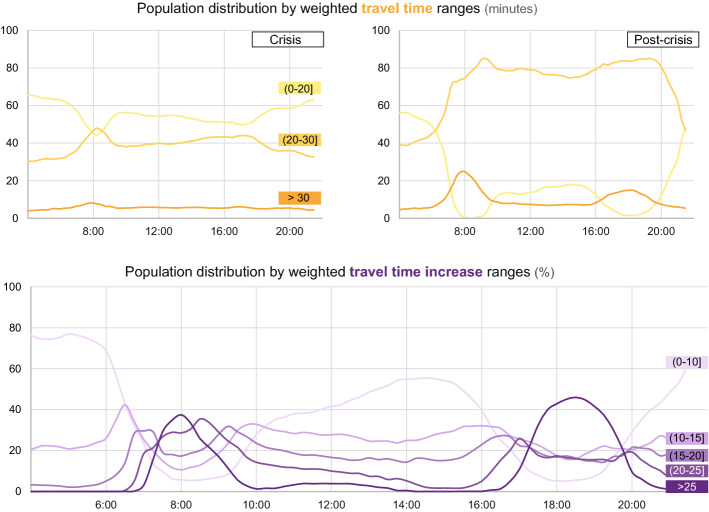
Population distribution by weighted travel time (**a**, **b**) and travel time increase (**c**)

The spatiotemporal differences found in the previous analysis are especially relevant when we consider the population affected. Figure 7 focuses on the share of the population (represented by the % of Twitter users) negatively affected by an increase in weighted travel times between the crisis and post-crisis periods. During the crisis period (Fig. 7a) and throughout the day, around 55% of the population share is in city areas. They have weighted travel times below 20 min, except for a brief period during the morning peak hour. Moreover, in the crisis, the share of the population facing WTT for over 30 min did not exceed 10% throughout the day. However, in the post-crisis period (Fig. 7b), the percentage of the population benefiting from a WTT below 20 min does not reach 20% during the daytime, descending to zero both in the morning and afternoon peak hours. Precisely in those moments, there is an increase in the population's share affected by weighted travel times of over 30 min. Apart from these remarks, in this post-crisis scenario, around 80% of the population share is distributed in Transport Zones with WTTs between 20 and 30 min during the daytime.

In analysing in detail the share of the population affected by different travel time increases between the crisis and post-crisis periods (Fig. 7c), the situation during the morning and afternoon peak hours is particularly interesting. In these times, over 40% of the population are affected by more than a 25% WTT increase between the crisis and post-crisis periods. However, although this increase in congestion is slightly higher at 8:00 h (21.9% and 19.1% respectively, see Fig. 4), a larger percentage of the population is affected during the afternoon. In addition, the situation at 12:00 h and lunch time, when the share of the population grows significantly in areas with the lowest range of WTT increase (> 10%) is remarkable. Comparing this with the map in Fig. 5 at this time (12:00 h scenario), we can deduce that, although the increase in WTT in the city centre remains at over 15–20%, the activity levels and population decrease.

Apart from that, congestion impacts in different areas of the city affect different population groups. The north–south dichotomy explained in previous travel time analyses is also visible in income level distribution, as Fig. 2 reveals. Members of the low-income population group that live in the southern residential areas of the city are most affected by the increase in traffic congestion during the morning, but they also suffer similar travel time increases when they have to return home in the afternoon. The concentration of activity and jobs in the northern part of the metropolitan area attracts workers every day from many other parts of the city. This increase in congestion could doubly affect the lower-income population. Apart from that, not only commuting-related mobility but also car-dependent access to schools might be key in these different spatial patterns between north and south.

All this is consistent with existing literature that suggests (1) congestion could be a by-product of economic development (Sweet 2014b) and the agglomeration of activities frequently gives rise to traffic congestion (Mondschein and Taylor 2017); (2) the lower-income population is more affected by economic recessions and recovery (Doran and Fingleton 2016; Lallement 2011), and therefore, by congestion increases, once confirmed the relation between congestion and economic cycles. Precisely, the income and travel times variables are included in the clustering analysis shown in the next section.

### Spatial typology: clustering analysis

Once the spatiotemporal differences between the two periods have been analysed, the next step is to define a spatial typology of Transport Zones in the Madrid metropolitan area. The overall variable statistic (R^2^) about each variable's impact in the grouping process in the clustering analysis shows that the variable *income* presents the lowest R2 value (0.59), while travel-time variables have R2 values 0.7 or higher. Therefore, the variable *income* is the less representative of all the variables considered, explaining better and putting into context the different groups.

**Fig. 8:**
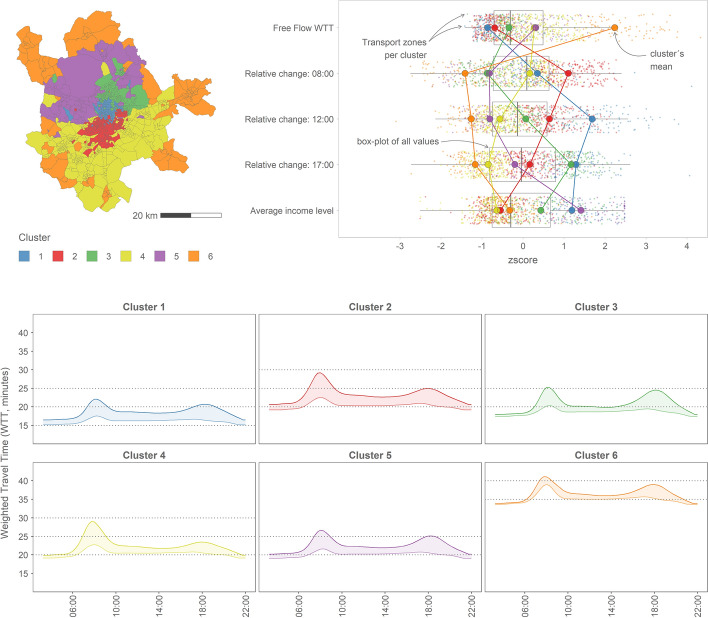
Spatial typology of Transport Zones in the Madrid metropolitan area

Figure 8 shows the six different clusters obtained in this analysis:

- Central areas in the city core (Cluster 1): This cluster encompasses the central mixed-use area of Madrid, with high income residents. In this case, travel times are very low (around 20 min average, Fig. 8c) and the increase in congestion is noticeable during both the peak hours in the morning and afternoon, but especially during the mornings’ non-peak hour (Fig. 8b). Comparing the trends of WTT in crisis and post-crisis periods (Fig. 8c), this cluster shows a similar performance in the two peak hours where the effects of congestion imply around a 5 min increase for both scenarios (25% growth compared to the crisis situation). In addition, while in the crisis afternoon rush hour, the peak is almost not visible, during the post-crisis the levels of congestion are just as important as those in the morning peak hour.
- Residential areas in the south of Madrid city (Cluster 2): In this case, because of the central location of the Transport Zones encompassed in this cluster, the travel times are low. Concerning the increase in congestion between crisis and post-crisis scenarios, these areas experienced a much more remarkable growth during the morning (Fig. 8b, this cluster presents the highest relative change at 8:00 h), affecting mainly low-income population. This is also shown in Fig. 8c, where the increase in travel time in the morning is the highest of all the clusters, around 7 min (reaching a 30% increase).
- Activity areas in the north of the city (Cluster 3): This cluster also presents very low travel times due to its central location. However, its performance is opposite to that of Cluster 2 in terms of congestion levels: these areas show a noticeable increase in travel times during the afternoon peak hour (Fig. 8b). These impacts of congestion can also be identified in Fig. 8c, presenting this cluster as the one with the highest increase in travel times during the post-crisis afternoon peak hour compared to travel times in the crisis period (a difference of more than 5 min or an increase of around 27%).
- Peri-urban zones in the metropolitan area (Clusters 4 and 5): These show high travel times and lower changes in congestion levels between crisis and post-crisis, compared to previous clusters. However, we can detect important differences between the north and south areas, with unequal consequences for particular groups of population:
  - Low-income residential zones in the south (Cluster 4): In this case, similarly but softer than cluster 2, these areas experience an important increase in congestion in the morning peak hour, while it is less notable in the afternoon. It is also remarkable that this cluster presents the lowest income levels of all the metropolitan areas (Fig. 8b).
  - High-income activity zones in the north (Cluster 5): On the contrary, this cluster includes the wealthiest areas of the metropolitan area and shows a higher increase of congestion in the afternoon (similar to the trend of Cluster 3).
- Rural areas in border municipalities (Cluster 6): These areas present completely different behaviour than the previous cases, with very long travel times and very low changes in the impact of congestion between periods.

## Conclusions

There is a direct relationship between the economic context and the use of cars in the city (Marshall and Dumbaugh 2020). Economic crises have an impact on mobility in general, both for mandatory (less trips to work/study) and non-mandatory trips (less leisure travel), particularly those affecting private vehicle rides: urban congestion decreases, and its side-effects are significantly reduced. This paper analyses changes in private vehicle travel times in two very different economic situations: a time of severe crisis and a post-crisis period. The analysis was conducted in Madrid, whose metropolitan area was severely affected by the economic crisis in Spain between 2008 and 2014, but the method proposed can be applied in any other metropolitan space.

The main results show how congestion levels increased in the morning and afternoon rush hours for both periods, but a more notable growth took place in the post-crisis period. The difference is also much more visible during the peak of the afternoon, which eventually was absent during the crisis. The results also show spatiotemporal differences among all the Transport Zones in the metropolitan area. In Madrid, different groups can be identified, allowing for the detection of city areas where travel time changes are much more noticeable at different times of the day. On the one hand, in the post-crisis situation, the areas in the south, those close to the city core and the peri-urban areas, show a higher increase in travel times due to higher congestion levels during the morning peak hour compared to the crisis period; while the northern parts of the metropolitan area show the opposite, with more significant growth during the afternoon. This effect is due to the differences related to land use and the location of activities: while most enterprises and jobs are located mainly in the northern areas, the south is mainly residential. On the other hand, the city core, where activity does not stop throughout the day, presents a more homogeneous congestion increase in all the temporal scenarios analysed, while the peripheral suburban zones of the metropolitan area show only a slight growth in average travel times when comparing crisis and post-crisis periods. These spatiotemporal traffic congestion patterns show the diverse impacts on populations living in different areas of the city.

A significant share of the population is affected by the high increase in congestion between the crisis and post-crisis periods: more than 40% of the population in the Madrid metropolitan area are affected by weighted travel times, which are 25% higher. Moreover, differences in land-use distribution generate many daily displacements south and north of people leaving their homes in the morning to get to work, and vice versa in the afternoon. This commuter pattern might affect most of the lower-income groups living in the southern residential neighbourhoods: they are affected the most by the increase in traffic congestion in the morning, but could also suffer a similar increase in travel times when they have to return home in the afternoon.

Previous studies have shown how European cities present different accessibility profiles during the day, regarding both the general trends and intensity of accessibility losses (Moya-Gómez and García-Palomares 2017). In well-known metropolitan areas such as Barcelona, Milano or Rome, the daily accessibility profiles are generally similar to Madrid. However, London or Paris presents higher congestion levels, and the peaks are more pronounced in the afternoon than in the morning. Understanding the effects of crisis and economic recovery situations on specific accessibility profiles in each city would require in-depth analysis for each case. Nevertheless, spatiotemporal patterns found for the Madrid metropolitan area are expected in other cases as well. Obviously, all the cities might experience congestion increases related to economic reactivation, but also congestion differences between central and peripheral areas, between residential and activity land uses or between higher and lower income areas.

Our research shows the extent to which new data sources provide an opportunity for assessing private vehicle accessibility and the impacts of urban congestion (Moya-Gómez and Geurs 2018; Zhao and Hu 2019). The recent availability of traffic data from commercial navigation companies (TomTom, Nokia Here, Garmin, among other) is a genuine leap forward for the study of dynamic travel times. Data about people’s locations during the day are becoming more spatially and temporally disaggregated (for example, geo-located tweets). In consequence, they contribute to greater detail in the land-use aspect of accessibility measures (Moya-Gómez et al. 2018), even though their use in accessibility analysis is still in its early stages (Condeço-Melhorado et al. 2018). The work developed in this paper is a good example of the potential for transportation studies the data provide. Big Data sources, such as TomTom, now offer datasets that address not only the analysis of spatiotemporal measures but also enable scholars to compare different periods, which allows us to assess how changes in the socio-economic context influence mobility patterns.

In view of the main aim of this paper and its scope, we considered a unidirectional relationship between the economic context, congestion and accessibility, although we are aware of the bidirectional causation, as revealed by the literature review. As the results of this paper show, economic recovery can lead to increased traffic congestion, with a consequential and dramatic increase in travel times, which can also impact economic activity (Marshall and Dumbaugh 2020). However, our paper has not included this possible effect of congestion on economic growth. In any case, understanding the changes in urban accessibility as a result of different socio-economic contexts is very useful for urban policymakers. Urban accessibility changes throughout the day because of different congestion levels, but the intensity of these changes depends on the mobility associated with each economic cycle. Identifying the impacts of these cycles, both through the general accessibility profile of the metropolitan area and, particularly, through those associated with different metropolitan zones, is very helpful for joint planning on binomial transport-land uses. The results presented in this paper show the strong impact the 2008 financial crisis had on accessibility in the Madrid metropolitan area. At present, with the development of the COVID-19 pandemic, we are facing an unprecedented global crisis, which will shape a new framework to which each urban area will need to adapt. The new redistribution of accessibility and its daily profiles will be a key element in the metropolitan transformations we are about to experience.

## Footnotes

1 Due to the crisis, unemployment rates reached 26% of the total active population, while the public budget balance decreased by 10.5% of GDP at the end of 2012. Source: Spanish National Institute of Statistics (INE).

2 https://developer.tomtom.com/traffic-stats.

3 It is a package of ArcGIS, which includes several tools for modelling and analysing transport networks, such as optimal route calculation, closest facility analysis, OD matrices, etc.

4 The 2013 version contains data on historical Speed Profiles for the years 2011 and 2012 while the 2018 version refers to the period between 2016 Q3 and 2018 Q2.

5 TomTom®’s FRC Definitions. FRC 0: Motorway, Freeway or other major road; FRC 1: Major road less important than a motorway; FRC 2: Other major road; FRC 3: Secondary road; FRC 4: Local connecting road; FRC 5: Local road of high importance; FRC 6: Local road; FRC 7: Local road of minor importance; FRC 8: Other roads.

6 Other economic variables, such as employment, were also considered but finally dismissed because the information was aggregated by municipality, making it difficult to implement such information as a mass factor in this study.

7 The 2016 data by census sections is aggregated as an average for each Transport Zone.

8 This tool performs a classification trying to find clusters among a specific sample of data using the k-means algorithm.
